# High Rates of Detection and Molecular Characterization of Porcine Adenovirus Serotype 5 (*Porcine mastadenovirus C*) from Diarrheic Pigs

**DOI:** 10.3390/pathogens11101210

**Published:** 2022-10-20

**Authors:** Kerry Gainor, Yussaira Castillo Fortuna, Angeline Steny Alakkaparambil, Wendy González, Yashpal Singh Malik, Souvik Ghosh

**Affiliations:** 1 Department of Biomedical Sciences, Ross University School of Veterinary Medicine, Basseterre P.O. Box 334, St. Kitts and Nevis, West Indies; KerryGainor@students.rossu.edu (K.G.); yussairacf@gmail.com (Y.C.F.); anju4nr@yahoo.com (A.S.A.); 2 Department of Biotechnology, School of Bio Sciences and Technology, Vellore Institute of Technology, Vellore 632014, India; 3 Epidemiological Surveillance Division, Dirección General de Ganadería, Santo Domingo 10410, Dominican Republic; wendygonz@gmail.com; 4 School of Veterinary Medicine, Faculty of Agronomic and Veterinary Sciences, Autonomous University of Santo Domingo, Calle Camino de Engombe 10904, Dominican Republic; 5 College of Animal Biotechnology, Guru Angad Dev Veterinary and Animal Science University, Ludhiana 141012, India; malikyps@gmail.com

**Keywords:** porcine adenovirus serotype 5, *Porcine mastadenovirus C*, pigs, diarrhea, DNA-dependent DNA polymerase (pol), hexon

## Abstract

Since the first report on isolation of porcine adenovirus serotype 5 (PAdV-5, species *Porcine mastadenovirus C* (*PAdV-C*)) from pigs with respiratory illness in Japan in 1987, PAdV-5 have been detected in a few fecal samples from healthy pigs and in some environmental samples. To date, only a single PAdV-5 strain (isolate HNF-70 from 1987) has been analyzed for the complete genome. We report here high detection rates of PAdV-5 (25.74%, 26/101 fecal samples) in diarrheic pigs at 3 different farms in the Caribbean country of Dominican Republic. After a long gap, the complete deduced amino acid sequences of the DNA-dependent DNA polymerase (pol) and hexon of two PAdV-5 strains (GES7 and Z11) were determined, revealing >99% sequence identities between PAdV-5 strains (HNF-70, GES7 and Z11) detected in different parts of the world and during different time periods (1987, and 2020–2021). By phylogenetic analysis, the putative hexon and pol of HNF-70, GES7 and Z11 exhibited similar clustering patterns, with the PAdV-5 strains forming a tight cluster near ruminant AdVs, distinct from the species *PAdV-A* and *-B*. GES7 and Z11 retained the various conserved features present in the putative pol and major late promoter region of HNF-70. Considering the paucity of data on current epidemiological status and genetic diversity of PAdV in porcine populations, our findings warrant similar studies on PAdV-5 and other PAdVs in clinically ill and healthy pigs. To our knowledge, this is the first report on detection and molecular characterization of PAdV-5 (*PAdV-C*) from diarrheic pigs.

## 1. Introduction

Porcine adenoviruses (PAdV) are double-stranded DNA viruses that belong to the genus *Mastadenovirus* within the family *Adenoviridae* [1]. To date, at least three species (*Porcine mastadenovirus A* (*PAdV-A*), *B* (*PAdV-B*) and *C* (*PAdV-C*)) and five serotypes (PAdV-1 to -5) have been recognized among PAdVs, with PAdV serotype 1-3, PAdV-4, and PAdV-5 belonging to species *PAdV-A*, *PAdV-B*, and *PAdV-C*, respectively [1,2]. Two additional PAdV serotypes (isolates GK/K92 and TG/K79) have also been proposed [3]. Based on analysis of the partial DNA-dependent DNA polymerase (pol)/hexon sequences, two PAdV strains, SVN1 and WI, were speculated to represent novel PAdV [4,5]. However, later, the partial sequences of SVN1 (hexon and pol) and WI (hexon) were found to be closely related to the recently available complete genome sequence of *PAdV-B* strain PAdV-B-HNU1 [6].

PAdV is considered as a low-grade porcine pathogen that rarely causes diseases of economic concern in domestic swine populations [2,7]. Since PAdVs are mostly associated with subclinical/asymptomatic infections, they (PAdV-3 and -5) have been used to develop viral vector vaccines against important porcine diseases and in gene transfer research [3,8]. PAdVs have been reported in healthy and clinically ill pigs [2,7]. In diseased pigs, PAdVs have been most commonly associated with diarrhea, although they have also been detected in animals with other clinical conditions (encephalitis, nephritis, respiratory disease, and reproductive disorders) [2]. Furthermore, PAdVs have been speculated to play roles in coinfections with economically important porcine pathogens [2,7]. Pigs infected with PAdV-4 and *Mycoplasma hyopneumoniae* were found to develop more severe pneumonia [2]. PAdVs have been detected in conjunction with *Rotavirus-A* (RVA) from diarrheic pigs [9]. Adenovirus inclusion bodies were more commonly observed in kidneys of pigs with *Porcine circovirus-2* (PCV2) associated disease [2].

Although PAdVs are believed to be endemic in porcine populations, epidemiological information on PAdVs is limited and primarily based on sero-surveillance studies conducted during the 1960–1980s [2,7,9,10,11]. As a result, the current global distribution of PAdV is not clear, and there is little data on PAdV-related mortality and morbidity rates in pigs [2,7,9]. Furthermore, the complete viral genome sequences, or full-length nucleotide (nt) sequences of important genes of only a handful of PAdV strains have been determined so far, which might not be sufficient to obtain conclusive insights into the evolution of PAdVs [1,3,6,12,13,14].

Among the PAdV serotypes (species), PAdV-1 and -3 (*PAdV-A*) have been associated with porcine gastrointestinal (GI) disease [2,7]. PAdV-4 (*PAdV-B*) has been reported in pigs with enteritis, encephalitis, nephritis, and pneumonia [2,6,7]. PAdV-5 (*PAdV-C*) was first isolated (isolates HNF-61 and -70) from nasal swabs of pigs exhibiting clinical signs (sneezing, nasal discharges, and coughing) of respiratory disease in Japan in 1987 [15]. A subsequent study by Kadoi et al. (1995) [16] had proposed another isolate (strain TG/K79, from the brain of a piglet that died shortly after) as a candidate of PAdV serotype 5. Since the study by Kadoi et al. (1995) was based on serological comparisons with viruses belonging to PAdV-1 to -4 and not PAdV-5 (the PAdV-5 isolates from 1987), it was concluded that TG/K79 might not represent PAdV-5 [3], although genomic evidence in support of this observation is lacking. To date, studies on PAdV-5 (*PAdV-C*) are very limited. Two different studies have identified PAdV-5 (3/5 fecal samples and <10% of 5 fecal-swab pools, respectively) in a few fecal samples from healthy pigs [17,18], whilst there are a few reports on detection of PAdV-5 in environmental samples (sewage effluent, water, and shellfish) [17,19,20,21,22], GenBank accession number KX570638]. The complete genomic sequence of only a single PAdV-5 strain (isolate HNF-70) has been analyzed so far [12]. In the present study, we report for the first-time high rates of detection and molecular characterization (full-length coding sequences (CDS) for the putative hexon and pol) of PAdV-5 in diarrheic pigs.

## 2. Results and Discussion

The present study was based on 101 fecal samples from diarrheic pigs at three different pig farms (Cabrera in Central-North, and Pedro Brand and Villa Mella in Central-South) in the Caribbean country of Dominican Republic (Supplementary Figure S1). Since the initial objective was to identify novel AdVs in diarrheic pigs, the porcine fecal samples were screened using a broad-range nested PCR screening assay (targeting a ~300 bp stretch of the AdV *pol* gene) that has been employed to amplify different genera of AdVs including novel AdVs [23,24]. It should be noted here that the AdV pan-*pol* nested PCR assay is based on a partial stretch of a single gene (*pol*) [23,24], and therefore, it might be possible that we missed detection of AdV positive samples that were not as similar to the target region. Twenty two of the 101 samples yielded the expected ~300 bp amplicon and were sequenced to confirm the presence of AdVs. However, by BLASTN (https://blast.ncbi.nlm.nih.gov/Blast, accessed on 27 July 2022) analysis, 14 of the sequences (~246 nt) shared maximum identities (>98%) with the *pol* of PAdV-5 (*PAdV-C*) isolate HNF-70, whilst the remaining 8 sequences shared maximum homology with non-AdV sequences, indicating non-specific amplification. This observation warrants the importance of sequencing-based confirmation of samples that test positive with AdV pan-*pol* nested PCR assays. Considering that all the 14 AdV sequences were closely related to PAdV-5, the negative samples (with the pan-*pol* nested PCR assay) were further screened using a PAdV-5-specific semi-nested PCR assay (supplementary Table S2), revealing an additional 12 PAdV-5 positive samples.

Taken together, we report here high rates of detection of PAdV-5 (25.74%, 26/101 samples) in diarrheic pigs. Although the sample size varied between farms, the PAdV-5 detection rates were higher on the farms in Cabrera (42.85%, 15/35) and Pedro Brand (37.5%, 6/16) compared to Villa Mella (10%, 5/50), which might be attributed to differences in animal husbandry practices between the farms (Table 1). The municipality of Cabrera (distance of ~165 km and ~180 km from Villa Mella and Pedro Brand, respectively) is located in the Central-Northern part of Dominican Republic, whilst Pedro Brand (~30 km from Villa Mella) and Villa Mella are located in the Central-Southern part of the country (Supplementary Figure S1). Since the pig farms in Cabrera, Pedro Brand and Villa Mella operate as independent units (no movement of animals, fodder, or equipment between the farms), potential transmission of PAdV-5 between the farms appeared to be unlikely. Our findings, although based on three different farms, provided preliminary evidence that PAdV-5 might be widely distributed in pig farms in the Dominican Republic, warranting large-scale molecular epidemiological studies on PAdV-5 in porcine populations throughout the country.

Among the different age groups of sampled pigs, the PAdV-5 detection rates were 23.52% (4/17), 11.42% (4/35), 55.55% (10/18), 0% (0/1), 30.76% (4/13), 14.28% (2/14) and 66.66% (2/3) in piglets, weaners, growers, gilt, farrow/pregnant sows, dry sows, and boars, respectively (Table 1). It has been proposed that PAdVs might play roles in coinfections [2]. AdV inclusion bodies were shown to be more common in kidneys of pigs infected with PCV2 [2]. In a study from Thailand, 18.4% (23/125) of diarrheic pigs tested positive for both PAdV and RVA [9]. As a part of another research project, the porcine fecal samples (from this study) were screened for the presence of PCV2 and RVA. PCV2 was detected in 20 (76.92%) of the 26 PAdV-5 positive samples, of which one animal died (a grower from Cabrera), whilst none of the diarrheic pigs tested positive for RVA (Table 1).

To date, only a single PAdV-5 strain (isolate HNF-70, AF289262) has been characterized for the complete viral genome (32621 bp in size) [12], whilst a 4199 bp sequence (containing open reading frames (ORF) 1 to 5) of PAdV-5 isolate HNF-61 (AF186621) and some PAdV-5 partial hexon sequences (~170 bp, or ~300 bp) are available in the GenBank database (https://www.ncbi.nlm.nih.gov/nuccore, accessed 27 July 2022). Except for isolates HNF-61 and HNF-70 (from pigs with respiratory illness in 1987) [12,15], no other PAdV-5 strain has been sequenced for the complete genome, or full-length CDS for important AdV proteins so far. In the present study, the partial *pol* sequences (~246 nt) of 12 (7, 3 and 2 strains from Cabrera, Pedro Brand and Villa Mella, respectively) of the 14 PAdV-5 strains (determined to confirm the results of the pan-*pol* nested PCR screening assay) were subjected to further analysis, whilst two of the sequences lacked high quality (Phred quality scores of <40). The partial deduced amino acid (aa) sequences (82 aa) of the putative pol of the 12 PAdV-5 strains shared 98.78–100% identities between themselves, and with that of the PAdV-5 reference strain HNF-70. Deduced aa identities of <80% were observed with cognate pol sequences of other AdVs. Phylogenetically, the partial pol sequences of the PAdV-5 strains from Cabrera, Pedro Brand and Villa Mella grouped with strain HNF-70 to form a single cluster, which was distinct from those of other mastadenoviruses (Supplementary Figure S3).

**Figure 1:**
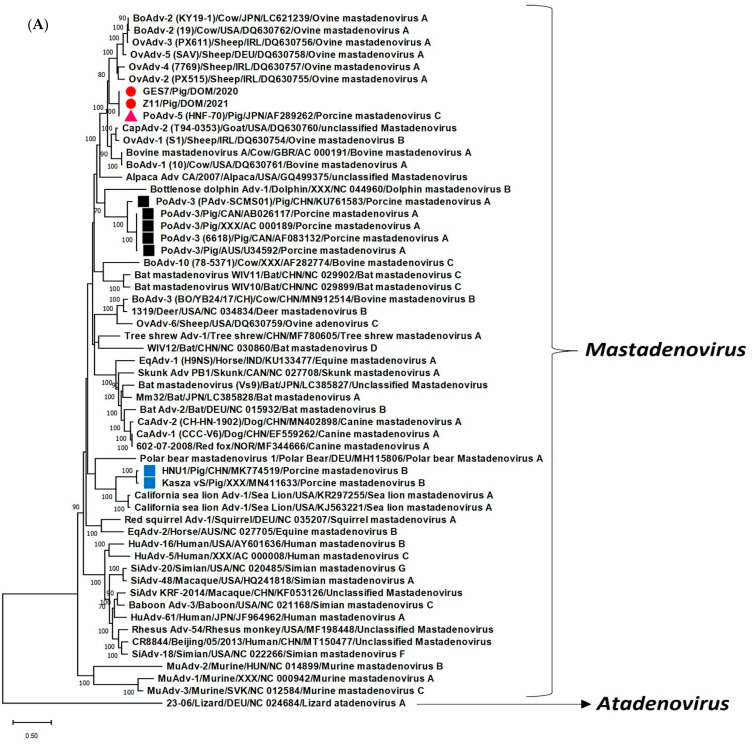

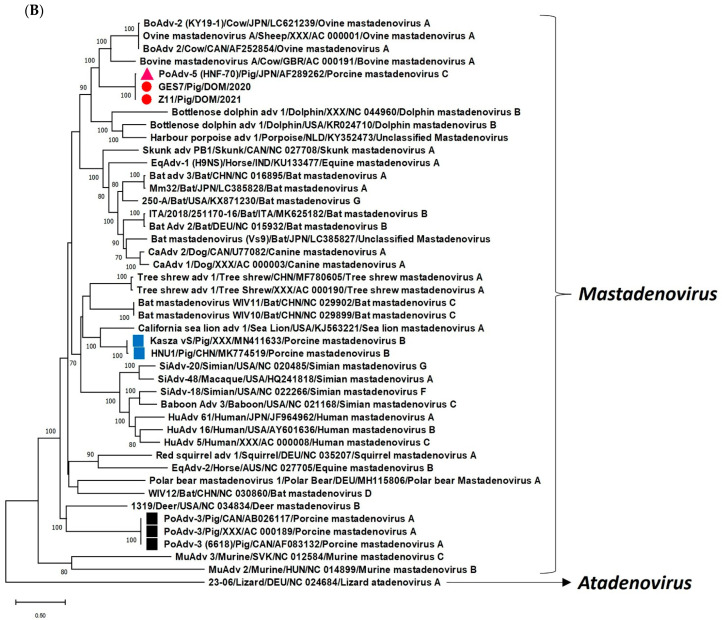
Phylogenetic analysis of the full-length deduced amino acid (aa) sequences of the putative hexon (**A**) and DNA-dependent DNA polymerase (pol) (**B**) of porcine adenovirus serotype 5 (PAdV-5) (species *Porcine mastadenovirus C* (*PAdV-C*)) strains GES7 and Z11 with those of other adenoviruses. The virus name/host/country/year are shown for GES7 and Z11, whilst the virus name (isolate name)/host/country/year/GenBank accession number/virus species have been mentioned for the other AdV strains. Red circles, a pink triangle, black, and blue squares indicate the PAdV-5 strains GES7 and Z11, the PAdV-5 reference strain (isolate HNF-70), viruses belonging to the species *PAdV-A*, and *-B*, respectively. A member of the genus *Atadenovirus* was used as the outgroup sequence. Bootstrap values < 70% are not shown. Scale bar, 0.5 substitutions per aa. Abbreviations: AdV, adenovirus; Bo, bovine; Ca, canine; Cap, caprine; Eq, equine; Hu, human; Mu, murine; Ov, ovine; PAdV, porcine adenovirus; Si, simian.

Since the partial pol sequences of the PAdV-5 strains from Cabrera, Pedro Brand and Villa Mella were closely related to each other (deduced aa identities of 98.78–100% between themselves, and phylogenetically, formed a tight cluster (Supplementary Figure S3)), we selected two samples (GES7 and Z11 from 2020 and 2021, respectively) that were only available in sufficient volumes for obtaining the full-length CDS for the putative AdV hexon and pol. The AdV hexon is the major viral capsid protein, which plays an important role in eliciting host immune responses [27], whilst the AdV pol participates in viral DNA replication [28]. Phylogenetic analysis of the pol constitutes an important basis for taxonomic classification of AdVs [1]. The complete deduced aa sequences of the putative hexon (910 aa) of GES7 and Z11 shared identities of 99.67% between themselves, and 99.78% and 99.89%, respectively, with that of PAdV-5 reference strain HNF-70 (Supplementary Figure S4), followed by identities of 82.46–85.92% with those of AdVs belonging to the species *Bovine mastadenovirus A* (*BAdV-A*), *caprine mastadenovirus* and *Ovine mastadenovirus A* (*OAdV-A*) and *B* (*OAdV-B*). Deduced aa identities of 73.19–75.88% and 65.25–65.38% were observed with the hexon of *PAdV-A* (PAdV-3) and *PAdV-B*, respectively. Phylogenetically, the hexon of GES7 and Z11 grouped with PAdV-5 isolate HNF-70 within a clade that consisted of two clusters of ruminant AdVs assigned to *BAdV-A*, *OAdV-A* and *OAdV-B* (Figure 1A).

The study on complete genomic analysis of PAdV-5 (isolate HNF-70) had analyzed the partial deduced aa sequence (843 aa, CDS: nt 6837-nt 4306) of the putative pol [12], although, later, the full-length pol sequence (1130 aa, CDS: join nt 12194-nt 12186, nt 7689-nt 4306) of HNF-70 was made available in the GenBank database (AC_000009). The pol (1130 aa) of GES7 and Z11 shared deduced aa identities of 99.91% between themselves, followed by identities of 99.56% and 99.47%, respectively, with that of HNF-70. With other AdVs, GES7 and Z11 shared <69% sequence identities. Low deduced aa identities (56.10–57.84% and 60.63–60.73%, respectively) were observed with the pol of *PAdV-A* and *PAdV-B* strains. By phylogenetic analysis, the pol of GES7 and Z11 clustered with HNF-70 near ruminant AdVs belonging to *BAdV-A* and *OAdV-A* (Figure 1B). GES7 and Z11 retained the conserved region I (YGDTDS) and the two putative zinc finger motifs that are conserved in the pol of PAdV-5 reference strain HNF-70 [12], although a single aa mismatch (V1019A) was observed between HNF-70 and the two study strains in one of the zinc finger motifs (Supplementary Figure S5). The inverted CAAT box, an upstream promoter element, the canonical TATA box, an initiator element and the 2 downstream activating elements, present in the putative major late promoter (MLP) region of HNF-70 [12], were also identified in GES7 and Z11 (Supplementary Figure S6).

Historically, PAdV-5 has been associated with porcine respiratory disease [2,7,15]. To our knowledge, the present study is the first report on high rates of detection (25.74%, 26/101) and molecular characterization of PAdV-5 (*PAdV-C*) from diarrheic pigs, although the virus has been previously identified in a few fecal samples from healthy pigs [17,18], indicating potential infection of the porcine GI tract. Since most of the PAdV-5 positive pigs were infected with PCV2 (associated with porcine enteritis [29]), and the samples were not screened for other enteric pathogens (except for PCV2 and RVA), we could not establish whether PAdV-5 caused GI disease in the pigs. After a long gap (more than 3 decades), the full-length CDS for two important PAdV-5 proteins (hexon and pol) were analyzed, revealing nearly absolute sequence identities between PAdV-5 strains (HNF-70, GES7 and Z11) detected in different parts of the world (Japan (Asia), and the Dominican Republic (Caribbean region)) and during different time periods (1987 and 2020-2021). Although phylogenetic analysis of the pol is crucial for taxonomic classification of AdVs [1], the PAdV-5 reference strain, HNF-70, was phylogenetically evaluated for only the hexon and pVIII proteins with a limited number of AdV sequences [12]. In the present study, the hexon and pol of HNF-70, GES7 and Z11 were subjected to phylogenetic analysis with a larger set of mastadenovirus sequences, revealing similar clustering patterns between the hexon and pol of PAdV-5 strains (Figure 1A,B), and corroborating previous observations that PAdV-5 (*PAdV-C*) is more related to ruminant AdVs (*BAdV-A*, *OAdV-A* and *-B*) than other AdVs, and distinct from members of the species *PAdV-A* and *-B* [12]. Considering the paucity of data on current epidemiological status and genetic diversity of PAdV in porcine populations [2,7,9], our findings warrant similar studies on PAdV-5 and other PAdVs in clinically ill and healthy pigs. The evolution of PAdV might be more complex than anticipated, as evident from evidence for recombination events involving the fiber genes of PAdV-5 and members of the genus *Atadenovirus* [30], underscoring the importance of analyzing the complete genomes, or at least the full-length sequences of important genes of several viral strains representing the different PAdV species.

## 3. Materials and Methods

### 3.1. Sampling

A total of 101 fecal samples were obtained from diarrheic pigs at three different farms in the Caribbean country of Dominican Republic (Supplementary Figure S1). Between August–November 2020, 35 and 16 fecal samples were collected on a pig farm in the municipality of Cabrera and Pedro Brand, respectively, and during January–February 2021, 50 fecal samples were obtained from a pig farm in the municipality of Villa Mella (Supplementary Figure S1). The samples were stored at −20 °C until further analysis. The Institutional Animal Care and Use Committee (IACUC) of the Ross University School of Veterinary Medicine (RUSVM), St. Kitts and Nevis, acknowledged the sampling process and use of the porcine fecal samples for the present study (RUSVM IACUC #: TSU6.10.22).

### 3.2. Amplification of Viral Genome

Viral DNA was extracted from the porcine fecal samples using the QIAamp Fast DNA Stool Mini Kit (Qiagen Sciences, Germantown, MD, USA) according to the manufacturer’s instructions. The samples were screened for adenoviral DNA using a broad-range nested PCR assay based on the *pol* gene, as described previously [23,24]. Samples that tested negative with the AdV pan-*pol* PCR assay were subjected to a PAdV-5-specific semi-nested PCR assay targeting the *pol* gene (Supplementary Table S2). Primers used in PCR/nested PCR assays to obtain the full-length hexon and pol CDS are shown in supplementary Table S2. Polymerase chain reactions were performed using the Platinum™ Taq DNA Polymerase (Invitrogen™, Thermo Fisher Scientific Corporation, Waltham, MA, USA) following the manufacturer’s instructions. Sterile water was used as the negative control in all PCR reactions.

### 3.3. Nucleotide Sequencing

The PCR amplicons were purified using the Wizard^®^ SV Gel and PCR Clean-Up kit (Promega, Madison, WI, USA) according to the instructions made available by the manufacturer. Nucleotide sequences were obtained using the ABI Prism Big Dye Terminator Cycle Sequencing Ready Reaction Kit on an ABI 3730XL Genetic Analyzer (Applied Biosystems, Foster City, CA, USA).

### 3.4. Sequence Analysis

The standard BLASTN and BLASTP program (Basic Local Alignment Search Tool, www.ncbi.nlm.nih.gov/blast, accessed on 27 July 2022) was used to perform homology searches for related nt and deduced aa sequences, respectively. The putative ORF coding for the PAdV-5 hexon protein was determined using the ORF finder (https://www.ncbi.nlm.nih.gov/orffinder/, accessed on 27 July 2022), whilst the putative CDS for pol were identified by alignment of the obtained nt sequences with the complete genome sequence of PAdV-5 reference strain HNF-70. Pairwise sequence identities were determined using the ‘align two or more sequences’ option of BLASTP (https://blast.ncbi.nlm.nih.gov/, accessed on 27 July 2022), or the EMBOSS Needle program (https://www.ebi.ac.uk/Tools/psa/emboss_needle/, accessed 27 July 2022). Multiple alignments of nt and deduced aa sequences were carried out using the Clustal Omega (https://www.ebi.ac.uk/Tools/msa/clustalo/, accessed 27 July 2022) program. Phylogenetic analysis was performed by the maximum likelihood (ML) method using the MEGA11 software [31], supported with 1000 bootstrap replicates and the LG + I + G model of substitution, as described previously [1].

### 3.5. GenBank Accession Numbers

The GenBank accession numbers for the PAdV-5 sequences determined in this study are OP087409-OP087414 and OP407941-OP407950.

## Figures and Tables

**Table 1:** Details of the fecal samples from diarrheic pigs that tested positive for porcine adenovirus 5 (species *Porcine mastadenovirus C*) in the Dominican Republic.

Animal/Sample Number	Age Group/Category of Animal ^1^	Location of the Pig Farm	Year of Sample Collection	Coinfection with	
				*Porcine circovirus 2* ^2^	*Rotavirus A* ^3^
DE10	Weaner	Pedro Brand ^4^	2020	Positive	Negative
DE22	Weaner	Cabrera ^4^	2020	Positive	Negative
DE92	Weaner	Pedro Brand	2020	Positive	Negative
ENG2	Grower	Cabrera	2020	Positive	Negative
ENG4	Grower	Cabrera	2020	Positive	Negative
ENG5	Grower	Cabrera	2020	Positive	Negative
ENG7	Grower	Cabrera	2020	Negative	Negative
ENG14	Grower	Cabrera	2020	Positive	Negative
ENG21	Grower	Cabrera	2020	Positive	Negative
ENG52	Grower	Cabrera	2020	Positive	Negative
GE4	Farrow ^5^ /Pregnant	Pedro Brand	2020	Negative	Negative
GE12	Farrow/Pregnant	Pedro Brand	2020	Negative	Negative
GES7	Farrow/Pregnant	Pedro Brand	2020	Positive	Negative
GES15	Farrow/Pregnant	Pedro Brand	2020	Positive	Negative
M8	Piglet	Cabrera	2020	Positive	Negative
MA1	Piglet	Cabrera	2020	Negative	Negative
MA5	Piglet	Cabrera	2020	Positive	Negative
MA9	Piglet	Cabrera	2020	Positive	Negative
N8	Grower	Cabrera	2020	Positive	Negative
VE8	Boar	Cabrera	2020	Positive	Negative
VE22	Boar	Cabrera	2020	Positive	Negative
D17	Weaner	Villa Mella ^4^	2021	Positive	Negative
PP3	Dry Sow	Villa Mella	2021	Negative	Negative
PP8	Dry Sow	Villa Mella	2021	Negative	Negative
Z11	Grower	Villa Mella	2021	Positive	Negative
Z13	Grower	Villa Mella	2021	Positive	Negative

^1^ Porcine age groups/animal categories as defined by the National Farm Animal Care Council (NFACC), Canada (https://www.nfacc.ca/codes-of-practice/pig-code#glossary, accessed 5 July 2022). ^2^ Fecal samples were screened for *Porcine circovirus 2* using a nested PCR assay targeting the replicase gene, as described previously [25]. ^3^ Screening for *Rotavirus A* (RVA) was carried out using a RVA VP6-specific RT-PCR assay, as reported in a previous study [26]. ^4^ Municipality in the Dominican Republic. ^5^ Refers to a sow that has recently given birth to piglets.

## Data Availability

Additional data is available as Supplementary Materials S1–S4.

## Supplementary Materials

The following supporting information can be downloaded at: https://www.mdpi.com/2076-0817/11/10/1210/s1, Supplementary Figure S1: The locations of the three porcine sampling sites in the Dominican Republic; Supplementary Table S2: Primers used in PCR assays to obtain the putative DNA-dependent DNA polymerase (pol) and hexon coding sequences of porcine adenovirus serotype 5 (PAdV-5, species *Porcine mastadenovirus C*) strains from the Dominican Republic; Supplementary Figure S3: Phylogenetic analysis of the partial deduced amino acid (aa) sequences (~82 aa) of the putative DNA-dependent DNA polymerase (pol) of porcine adenovirus serotype 5 (PAdV-5) (species *Porcine mastadenovirus C* (*PAdV-C*)) strains detected on the three pig farms in Dominican Republic (DOM) with those of other adenoviruses; Supplementary Figure S4: Multiple alignment of the putative hexon proteins of porcine adenovirus (PAdV) strains GES7 and Z11 with that of PAdV serotype 5 (PAdV-5) (species *Porcine mastadenovirus C*) reference strain HNF-70; Supplementary Figure S5: Multiple alignment of the putative DNA-dependent polymerase (pol) proteins of porcine adenovirus (PAdV) strains GES7 and Z11 with that of PAdV serotype 5 (PAdV-5) (species *Porcine mastadenovirus C*) reference strain HNF-70; Supplementary Figure S6: Identification of the inverted CAAT box (shown with green), an upstream promoter element (blue), the canonical TATA box (orange), an initiator element (yellow), and the 2 downstream activating elements (grey and pink) in the putative major late promoter region of porcine adenovirus serotype 5 (species *Porcine mastadenovirus C*) strains HNF-70, GES7 and Z11.
