# Supplementary material for: High Rates of Detection and Molecular Characterization of Porcine Adenovirus Serotype 5 (Porcine mastadenovirus C) from Diarrheic Pigs
Source: Pathogens. 2022 Oct 20;11(10):1210. doi: 10.3390/pathogens11101210 (PMC9610507; doi:10.3390/pathogens11101210)
Supplement: Supplementary file 1 [file pathogens-11-01210-s001.zip › Supplementary figure S1.pdf]

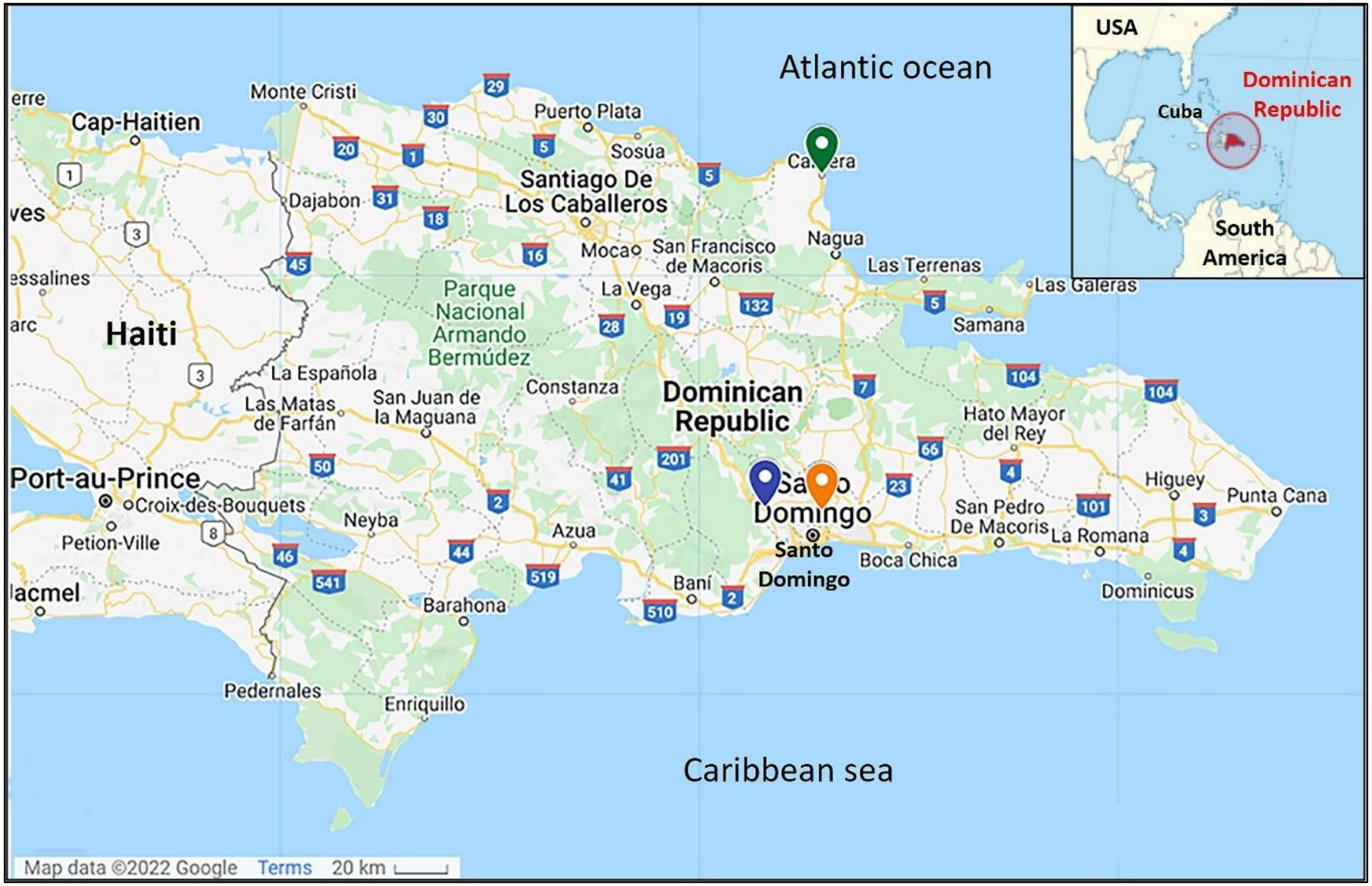

Supplementary figure S1. The locations of the three porcine sampling sites in the Dominican Republic. The pig farm in the municipality of Cabrera, Pedro Brand, and Villa Mella is indicated with green, blue, and orange pins, respectively. The map of Dominican Republic was adapted from https://www.google.com/maps (accessed June 2, 2022). ***Inset:*** Geographical location of the Caribbean nation of Dominican Republic (encircled and highlighted with red). The map was adapted from https://commons.wikimedia.org/wiki/File:Dominican_Republic_in_the_world_(W3).svg (author: TUBS, https://commons.wikimedia.org/wiki/User:TUBS) on June 2, 2022, and is licensed for free sharing and adaptation under the Creative Commons Attribution-Share Alike 3.0 Unported license.
