# Supplementary material for: High Rates of Detection and Molecular Characterization of Porcine Adenovirus Serotype 5 (Porcine mastadenovirus C) from Diarrheic Pigs
Source: Pathogens. 2022 Oct 20;11(10):1210. doi: 10.3390/pathogens11101210 (PMC9610507; doi:10.3390/pathogens11101210)

Supplementary figure S3. Phylogenetic analysis of the partial deduced amino acid (aa) sequences (~82 aa) of the putative DNA-dependent DNA polymerase (pol) of porcine adenovirus serotype 5 (PAdV-5) (species *Porcine mastadenovirus C* (*PAdV-C*)) strains detected on the three pig farms in Dominican Republic (DOM) with those of other adenoviruses. The tree was constructed by MEGA11 software using the neighbor-joining method with the Poisson model of substitution, Gamma distributed (G), and 1000 bootstrap replicates. The DOM PAdV-5 strains exhibited similar clustering patterns with other mathematical models of substitution, such as the p-distance model, equal input model, Dayhoff model, and Jones, Taylor, Thornton (JTT) model. The virus name/host/country/year are shown for the DOM PAdV-5 strains, whilst the virus name (isolate name)/host/country/year/GenBank accession number/virus species have been mentioned for the other AdV strains. Red, blue, and green circles indicate the DOM PAdV-5 strains from Cabrera, Pedro Brand, and Villa Mella, respectively. The PAdV-5 reference strain (isolate HNF-70), viruses belonging to the species *PAdV-A*, and *-B*, have been highlighted with a pink triangle, brown, and grey squares, respectively. A member of the genus *Atadenovirus* was used as the outgroup sequence. Bootstrap values < 70% are not shown. Scale bar, 0.5 substitutions per aa. Abbreviations: AdV, adenovirus; Bo, bovine; Ca, canine; Cap, caprine; Eq, equine; Hu, human; Mu, murine; Ov, ovine; PAdV, porcine adenovirus; Si, simian.

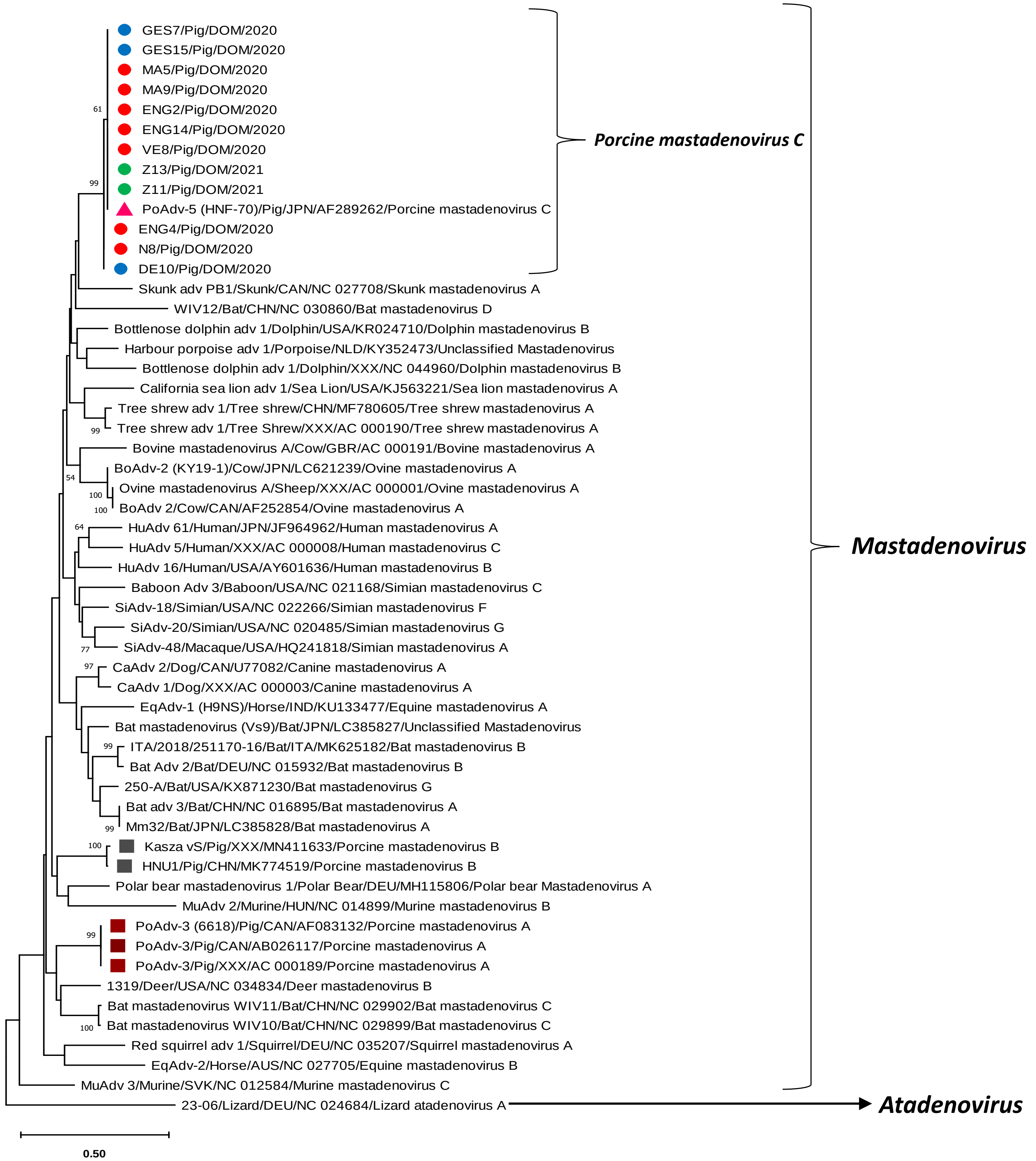

Supplement: Supplementary file 1 [file pathogens-11-01210-s001.zip › Supplementary figure S3.pdf]
