# Supplementary material for: High Rates of Detection and Molecular Characterization of Porcine Adenovirus Serotype 5 (Porcine mastadenovirus C) from Diarrheic Pigs
Source: Pathogens. 2022 Oct 20;11(10):1210. doi: 10.3390/pathogens11101210 (PMC9610507; doi:10.3390/pathogens11101210)
Supplement: Supplementary file 1 [file pathogens-11-01210-s001.zip › Supplementary figure S4.pdf]

Supplementary figure S4. Multiple alignment of the putative hexon proteins of porcine adenovirus (PAdV) strains GES7 and Z11 with that of PAdV serotype 5 (PAdV-5) (species *Porcine mastadenovirus C*) reference strain HNF-70. Amino acid (aa) mismatches are highlighted with green. The virus serotype (isolate)/GenBank accession number are shown for PAdV-5 isolate HNF-70, whilst the virus name/host/country/year have been mentioned for strains GES7 and Z11. A '*' denotes an identical aa residue. Numbers to the right indicate the positions of the aa for respective PAdV strains.

```
PAdV-5_(HNF-70)/AF289262      MATPSMMPQWSYMHIAGQDASEYLSPGLVQFAQATETYFKLGNKFRNPTVAPTHDVTTER    60
GES7/Pig/DOM/2020             MATPSMMPQWSYMHIAGQDASEYLSPGLVQFAQATETYFKLGNKFRNPTVAPTHDVTTER    60
Z11/Pig/DOM/2021              MATPSMMPQWSYMHIAGQDASEYLSPGLVQFAQATETYFKLGNKFRNPTVAPTHDVTTER    60
                              ************************************************************

PAdV-5_(HNF-70)/AF289262      SQRLQLRFVPVDREDTQYTYKTRFQLAVGDNRVLDMASTYFDIRGVIDRGPSFKPYSGTA    120
GES7/Pig/DOM/2020             SQRLQLRFVPVDREDTQYTYKTRFQLAVGDNRVLDMASTYFDIRGVIDRGPSFKPYSGTA    120
Z11/Pig/DOM/2021              SQRLQLRFVPVDREDTQYTYKTRFQLAVGDNRVLDMASTYFDIRGVIDRGPSFKPYSGTA    120
                              ************************************************************

PAdV-5_(HNF-70)/AF289262      YNPLAPKASVNNTMFENSNNPDQIRSMAQASFATPINDDTGAIEIPNQIIDLNYQPEPQL    180
GES7/Pig/DOM/2020             YNPLAPKASVNNTMFENSNNPDQIRSMAQASFATPINDDTGAIEIPNQIIDLNYQPEPQL    180
Z11/Pig/DOM/2021              YNPLAPKASVNNTMFENSNNPDQIRSMAQASFATPINDDTGAIEIPNQIIDLNYQPEPQL    180
                              ************************************************************

PAdV-5_(HNF-70)/AF289262      GEESWVSDVIDKPTQAAGRILSANNDPIPCYGSYARPTNQNGGQATAAVETINFKAGNAA    240
GES7/Pig/DOM/2020             GEESWVSDVIDKPTQAAGRILSANNDPIPCYGSYARPTNQNGGQATAAVETINFKAGNAA    240
Z11/Pig/DOM/2021              GEESWVSDVIDKPTQAAGRILSANNDPIPCYGSYARPTNQNGGQATAAVETINFKAGNAA    240
                              ************************************************************

PAdV-5_(HNF-70)/AF289262      GIPDTGFVMEDVNLTAPDTHLVYKVSEEDAGKTPALGQQAAPNRANYIGFRDNFIGLMYY    300
GES7/Pig/DOM/2020             GIPDTGFVMEDVNLTAPDTHLVYKVSEEDAGKTPALGQQAAPNRANYIGFRDNFIGLMYY    300
Z11/Pig/DOM/2021              GIPDTGFVMEDVNLTAPDTHLVYKVSEEDAGKTPALGQQAAPNRANYIGFRDNFIGLMYY    300
                              ************************************************************

PAdV-5_(HNF-70)/AF289262      NSNGNLGVLAGQASQLNAVVDLQDRNTELSYQLMLDSLYDRSRYFSMWNQAIDSYDQDVR    360
GES7/Pig/DOM/2020             NSNGNLGVLAGQASQLNAVVDLQDRNTELSYQLMLDSLYDRSRYFSMWNQAIDSYDQDVR    360
Z11/Pig/DOM/2021              NSNGNLGVLAGQASQLNAVVDLQDRNTELSYQLMLDSLYDRSRYFSMWNQAIDSYDQDVR    360
                              ************************************************************

PAdV-5_(HNF-70)/AF289262      VIENNGVEDDMPNYCFPLSGINTGATSVQVSLNQNNWVATNGASVNNYINIGNLDCMEIN    420
GES7/Pig/DOM/2020             VIENNGVEDDMPNYCFPLSGINTGATSVQVSLNQNNWVATNGASVNNYINIGNLDCMEIN    420
Z11/Pig/DOM/2021              VIENNGVEDDMPNYCFPLSGINTGATSVQVSLNQNNWVATNGASVNNYINIGNLDCMEIN    420
                              ************************************************************

PAdV-5_(HNF-70)/AF289262      LAANLWRGFLYSNIALYLPDDLKFTPPNVVLPENTNTYAYMNGRLPAGGLVDTYVNIGAR    480
GES7/Pig/DOM/2020             LAANLWRGFLYSNIALYLPDDLKFTPPNVVLPENTNTYAYMNGRLPAGGLVDTYVNIGAR    480
Z11/Pig/DOM/2021              LAANLWRGFLYSNIALYLPDDLKFTPPNVVLPENTNTYAYMNGRLPAGGLVDTYVNIGAR    480
                              ************************************************************

PAdV-5_(HNF-70)/AF289262      WSLDVMDNVNPFNHHRNAGLRYRSQLLGNGRYCQFHIQVPQKFFAIRNLLLLPGTYTYEW    540
GES7/Pig/DOM/2020             WSLDVMDNVNPFNHHRNAGLRYRSQLLGNGRYCQFHIQVPQKFFAIRNLLLLPGTYTYEW    540
Z11/Pig/DOM/2021              WSLDVMDNVNPFNHHRNAGLRYRSQLLGNGRYCQFHIQVPQKFFAIRNLLLLPGTYTYEW    540
                              ************************************************************

PAdV-5_(HNF-70)/AF289262      SFRKDVNMVLQSTLGNDLRVDGASIRIDSVNLYASFFPMAHNTASTLEAMLRNDTNDQSF    600
GES7/Pig/DOM/2020             SFRKDVNMVLQSTLGNDLRVDGASIRIDSVNLYASFFPMAHNTASTLEAMLRNDTNDQSF    600
Z11/Pig/DOM/2021              SFRKDVNMVLQSTLGNDLRVDGASIRIDSVNLYASFFPMAHNTASTLEAMLRNDTNDQSF    600
                              ************************************************************

PAdV-5_(HNF-70)/AF289262      IDYLSSANMLYPIPAGASNLPISIPSRNWAAFRGWSFTRLKQRETPALGSPFDPYFTYSG    660
GES7/Pig/DOM/2020             IDYLSSANMLYPIPAGASNLPIFIPSRNWAAFRGWSFTRLKQRETPALGSPFDPYFTYSG    660
Z11/Pig/DOM/2021              IDYLSSANMLYPIPAGASNLPISIPSRNWAAFRGWSFTRLKQRETPALGSPFDPYFTYSG    660
                              ********************** *************************************

PAdV-5_(HNF-70)/AF289262      TIPYLDGTFYLNHTFRRVSIQFDSSVQWPGNDRLLTPNEFEIKRTVDGEGYTVAQSNMTK    720
GES7/Pig/DOM/2020             TIPYLDGTFYLNHTFRRVSIQFDSSVQWPGNDRLLTPNEFKIKRTVDGEGYTVAQSNMTK    720
Z11/Pig/DOM/2021              TIPYLDGTFYLNHTFRRVSIQFDSSVQWPGNDRLLTPNEFEIKRTVDGEGYTVAQSNMTK    720
                              **************************************** *******************
```

```
PAdV-5_(HNF-70)/AF289262       DWFLVQMLANYNIGYQGYHLPDGYKDRNYSFLRNFQPMCRQVVDTANYAAYQNVMLTNQH    780
GES7/Pig/DOM/2020              DWFLVQMLANYNIGYQGYHLPDGYKDRNYSFLRNFQPMCRQVVDTANYAAYQNVMLTNQH    780
Z11/Pig/DOM/2021               DWFLVQMLANYNIGYQGYHLPDGYKDRNYSFLRNFQPMCRQVVDTANYAAYQNVMLTNQH    780
                               ************************************************************

PAdV-5_(HNF-70)/AF289262       NNSGFSGFASAALPREGHPYPANWPYPLVGANAVPTITQRKFLCDRTMWRIPFSSNFMSM    840
GES7/Pig/DOM/2020              NNSGFSGFASAALPREGHPYPANWPYPLVGANAVPTITQRKFLCDRTMWRIPFSSNFMSM    840
Z11/Pig/DOM/2021               NNSGFSGFASAALPREGHPYAANWPYPLVGANAVPTITQRKFLCDRTMWRIPFSSNFMSM    840
                               ******************** ***************************************

PAdV-5_(HNF-70)/AF289262       GSLTDLGQNLLYANSAHALDMTFEVDAMEEPTLLYILFEVFDLVRVHQPHRGVIETVYLR    900
GES7/Pig/DOM/2020              GSLTDLGQNLLYANSAHALDMTFEVDAMEEPTLLYILFEVFDLVRVHQPHRGVIETVYLR    900
Z11/Pig/DOM/2021               GSLTDLGQNLLYANSAHALDMTFEVDAMEEPTLLYILFEVFDLVRVHQPHRGVIETVYLR    900
                               ************************************************************

PAdV-5_(HNF-70)/AF289262       TPFSAGNATT      910
GES7/Pig/DOM/2020              TPFSAGNATT      910
Z11/Pig/DOM/2021               TPFSAGNATT      910
                               **********
```
