# Supplementary material for: High Rates of Detection and Molecular Characterization of Porcine Adenovirus Serotype 5 (Porcine mastadenovirus C) from Diarrheic Pigs
Source: Pathogens. 2022 Oct 20;11(10):1210. doi: 10.3390/pathogens11101210 (PMC9610507; doi:10.3390/pathogens11101210)
Supplement: Supplementary file 1 [file pathogens-11-01210-s001.zip › Supplementary figure S5.pdf]

Supplementary figure S5. Multiple alignment of the putative DNA-dependent polymerase (pol) proteins of porcine adenovirus (PAdV) strains GES7 and Z11 with that of PAdV serotype 5 (PAdV-5) (species *Porcine mastadenovirus C*) reference strain HNF-70. Amino acid (aa) mismatches are highlighted with green. The two putative zinc finger motifs are shown with underline and red font, whilst the conserved region I has been highlighted with blue. The virus serotype (isolate)/GenBank accession number are shown for PAdV-5 isolate HNF-70, whilst the virus name/host/country/year have been mentioned for strains GES7 and Z11. A '*' denotes an identical aa residue. Numbers to the right indicate the positions of the aa for respective PAdV strains.

```
PAdV-5_(HNF-70)/AF289262      MSVVPSDGSGRLLGAEGHPKHQQKGRGHGDEAETGRQTYAGTARGRPARSGPVRGNLVAK    60
GES7/Pig/DOM/2020             MSVVPSDGSGRLLGAEGHPKHQQKGRGHGDEAETGRQTSAGTARGRPARSGPVRGNLVAK    60
Z11/Pig/DOM/2021              MSVVPSDGSGRLLGAEGHPKHQQKGRGHGDEAETGRQTSAGTARGRPARSGPVRGNLVAK    60
                              ************************************** *********************

PAdV-5_(HNF-70)/AF289262      RATLSASGTLEDGTHVDIKFHSKTLEALENLFHLHLLQLPPLPPISTSNVSDVLQLLAPR    120
GES7/Pig/DOM/2020             RATLSASGTLEDGTHVDIKFHSKTLEALENLFHLHLLQLPPLPPISTSNVSDVLQLLSPR    120
Z11/Pig/DOM/2021              RATLSASGTLEDGTHVDIKFHSKTLEALENLFHLHLLQLPPLPPISTSNVSDVLQLLSPR    120
                              ********************************************************* **

PAdV-5_(HNF-70)/AF289262      EGSVVVYNRGRVSVKQVEIREPKAHFDIQFLLKQGKVYLIKEIQDTQKCEYCGDFFKTSH    180
GES7/Pig/DOM/2020             EGSVVAYNRGRVSVKQVEIREPKTHFDIQFLLKQGKVYLIKEIQDTQKCEYCGDFFKTSH    180
Z11/Pig/DOM/2021              EGSVVAYNRGRVSVKQVEIREPKTHFDIQFLLKQGKVYLIKEIQDTQKCEYCGDFFKTSH    180
                              ***** ***************** ************************************

PAdV-5_(HNF-70)/AF289262      TCSVRRRDFYFHHVHHKSSDWWENIPFQPLGSYRETERLYITYDVETYTWHGKHGKQLVP    240
GES7/Pig/DOM/2020             TCSVRRRDFYFHHVHHKSSDWWENIPFQPLGSYRETERLYITYDVETYTWHGKHGKQLVP    240
Z11/Pig/DOM/2021              TCSVRRRDFYFHHVHHKSSDWWENIPFQPLGSYRETERLYITYDVETYTWHGKHGKQLVP    240
                              ************************************************************

PAdV-5_(HNF-70)/AF289262      FLLVFHISGEPHLVKVAESVAQDLNWCPWTEKHTFYYLNPQKSAVGRMFKSFRDELQNRV    300
GES7/Pig/DOM/2020             FLLVFHISGEPHLVKVAESVAQDLNWCPWTEKHTFYYLNPQKSAVGRMFKSFRDELQNRV    300
Z11/Pig/DOM/2021              FLLVFHISGEPHLVKVAESVAQDLNWCPWTEKHTFYYLNPQKSAVGRMFKSFRDELQNRV    300
                              ************************************************************

PAdV-5_(HNF-70)/AF289262      TQDLWKTFLSQNPHLVETAARLNLGSVDDITPEVLKKEKICGEPRFIEVYVIGHNISGFD    360
GES7/Pig/DOM/2020             TQDLWKTFLSQNPHLVETAARLNLGSVDDITPEVLKKEKICGEPRFIEVYVIGHNISGFD    360
Z11/Pig/DOM/2021              TQDLWKTFLSQNPHLVETAARLNLGSVDDITPEVLKKEKICGEPRFIEVYVIGHNISGFD    360
                              ************************************************************

PAdV-5_(HNF-70)/AF289262      EIVLAAQVICHQSKTIKAFKISRNFMPRNGKILFNDITFGLPNPLFEKRKEFQEWERGSM    420
GES7/Pig/DOM/2020             EIVLAAQVICHQSKTIKAFKISRNFMPRNGKILFNDITFGLPNPLFEKRKEFQEWERGSM    420
Z11/Pig/DOM/2021              EIVLAAQVICHQSKTIKAFKISRNFMPRNGKILFNDITFGLPNPLFEKRKEFQEWERGSM    420
                              ************************************************************

PAdV-5_(HNF-70)/AF289262      AVQDMKQQFVKLMVRDTLMLTHTSLRNAAKAYDLPVEKGCCPYEAVNEFYRTGTYQKDED    480
GES7/Pig/DOM/2020             AVQDMKQQFVKLMVRDTLMLTHTSLRNAAKAYDLPVEKGCCPYEAVNEFYRTGTYQKDED    480
Z11/Pig/DOM/2021              TVQDMKQQFVKLMVRDTLMLTHTSLRNAAKAYDLPVEKGCCPYEAVNEFYRTGTYQKDED    480
                               ***********************************************************

PAdV-5_(HNF-70)/AF289262      GFPSLRYWKDQEEYALNKELWREKKCGAYDLISSTLTYCAQDVLVTSSLVRKLQESYQSF    540
GES7/Pig/DOM/2020             GFPSLRYWKDQEEYALNKELWREKKCGAYDLISSTLTYCAQDVLVTSSLVRKLQESYQSF    540
Z11/Pig/DOM/2021              GFPSLRYWKDQEEYALNKELWREKKCGAYDLISSTLTYCAQDVLVTSSLVRKLQESYQSF    540
                              ************************************************************

PAdV-5_(HNF-70)/AF289262      IANEVNLPDSSFNIFQRPTISSNSHAIFKQILYRAEKPERQHLGEVLLAPSNEMYDYVRQ    600
GES7/Pig/DOM/2020             IANEVNLPDSSFNIFQRPTISSNSHAIFKQILYRAEKPERQHLGEVLLAPSNEMYDYVRQ    600
Z11/Pig/DOM/2021              IANEVNLPDSSFNIFQRPTISSNSHAIFKQILYRAEKPERQHLGEVLLAPSNEMYDYVRQ    600
                              ************************************************************

PAdV-5_(HNF-70)/AF289262      SIRGGRCYPTYIGILHEPIYVYDICGMYASALTHPMPSGSPLNPFERALAVAVWEDQLKS    660
GES7/Pig/DOM/2020             SIRGGRCYPTYIGILHEPIYVYDICGMYASALTHPMPSGSPLNPFERALAVAVWEDQLKS    660
Z11/Pig/DOM/2021              SIRGGRCYPTYIGILHEPIYVYDICGMYASALTHPMPSGSPLNPFERALAVAVWEDQLKS    660
                              ************************************************************

PAdV-5_(HNF-70)/AF289262      VGQKMDYFDEKLLPGIFTIDADPPDESFLDVLPPFCSRKGGRLCWTNEPLRGEVATSVDV    720
GES7/Pig/DOM/2020             VGQKMDYFDEKLLPGIFTIDADPPDESFLDVLPPFCSRKGGRLCWTNEPLRGEVATSVDV    720
Z11/Pig/DOM/2021              VGQKMDYFDEKLLPGIFTIDADPPDESFLDVLPPFCSRKGGRLCWTNEPLRGEVATSVDV    720
                              ************************************************************
```

```
PAdV-5_(HNF-70)/AF289262      ITLHNRGWRVRLVPDERTTIFPEWKCLAKEYVQLNIAAKEKADREKNQTMRSIAKLLSNA    780
GES7/Pig/DOM/2020             ITLHNRGWRVRLVPDERTTIFPEWKCLAKEYVQLNIAAKEKADREKNQTMRSIAKLLSNA    780
Z11/Pig/DOM/2021              ITLHNRGWRVRLVPDERTTIFPEWKCLAKEYVQLNIAAKEKADREKNQTMRSIAKLLSNA    780
                              ************************************************************

PAdV-5_(HNF-70)/AF289262      LYGSFATRLDNKKIVFSDQLEEESSKNISRGKYSVKSSSFIETDNFSADIMPEFVVAYPP    840
GES7/Pig/DOM/2020             LYGSFATRLDNKKIVFSDQLEEESSKNISRGKYSVKSSSFIETDNFSADIMPEFVVAYPP    840
Z11/Pig/DOM/2021              LYGSFATRLDNKKIVFSDQLEEESSKNISRGKYSVKSSSFIETDNFSADIMPEFVVAYPP    840
                              ************************************************************

PAdV-5_(HNF-70)/AF289262      VADVSNEDNEAAEEATPFIGKSDHVTYKYKPITFLDVEDDDVCLHTLESSSSLVLNNRYA    900
GES7/Pig/DOM/2020             VADVSNEDNEAAEEATPFIGKSDHVTYKYKPITFLDVEDDDVCLHTLESSSSLVLNNRYA    900
Z11/Pig/DOM/2021              VADVSNEDNEAAEEATPFIGKSDHVTYKYKPITFLDVEDDDVCLHTLESSSSLVLNNRYA    900
                              ************************************************************

PAdV-5_(HNF-70)/AF289262      SHLASFVLAWARVFVSEWSEFLYENDRGVPMEERQIKSVYGDTDSLFVTEEGHRLMKEKG    960
GES7/Pig/DOM/2020             SHLASFVLAWARVFVSEWSEFLYENDRGVPMEERQIKSVYGDTDSLFVTEEGHRLMKEKG    960
Z11/Pig/DOM/2021              SHLASFVLAWARVFVSEWSEFLYENDRGVPMEERQIKSVYGDTDSLFVTEEGHRLMKEKG    960
                              ************************************************************

PAdV-5_(HNF-70)/AF289262      KHRIKKNGGSLVFDPQHPQVTWLVECETRCDKCGEDAYSPTSVFLAPKLYALKSTVCSVC    1020
GES7/Pig/DOM/2020             KHRIKKNGGSLVFDPQHPQVTWLVECETRCDKCGEDAYSPTSVFLAPKLYALKSTVCSAC    1020
Z11/Pig/DOM/2021              KHRIKKNGGSLVFDPQHPQVTWLVECETRCDKCGEDAYSPTSVFLAPKLYALKSTVCSAC    1020
                              ********************************************************** *

PAdV-5_(HNF-70)/AF289262      GYVGKGKLRAKGHATSELSFDVLQRCYLEDLQLGSEKFKTSRLSLKRTLASCQSNAAPFT    1080
GES7/Pig/DOM/2020             GYVGKGKLRAKGHATSELSFDVLQRCYLEDLQLGSEKFKTSRLSLKRTLASCQSNAAPFT    1080
Z11/Pig/DOM/2021              GYVGKGKLRAKGHATSELSFDVLQRCYLEDLQLGSEKFKTSRLSLKRTLASCQSNAAPFT    1080
                              ************************************************************

PAdV-5_(HNF-70)/AF289262      VTEATLTRTLRPWKDKTLTQIDQNRLIPYSTSRPNPRNTDLCWMTLPWDS    1130
GES7/Pig/DOM/2020             VTEATLTRTLRPWKDKTLTQIDQNRLIPYSTSRPNPRNTDLCWMTLPWDS    1130
Z11/Pig/DOM/2021              VTEATLTRTLRPWKDKTLTQIDQNRLIPYSTSRPNPRNTDLCWMTLPWDS    1130
                              **************************************************
```
