# Supplementary material for: High Rates of Detection and Molecular Characterization of Porcine Adenovirus Serotype 5 (Porcine mastadenovirus C) from Diarrheic Pigs
Source: Pathogens. 2022 Oct 20;11(10):1210. doi: 10.3390/pathogens11101210 (PMC9610507; doi:10.3390/pathogens11101210)
Supplement: Supplementary file 1 [file pathogens-11-01210-s001.zip › Supplementary figure S6.pdf]

Supplementary figure S6. Identification of the inverted CAAT box (shown with green), an upstream promoter element (blue), the canonical TATA box (orange), an initiator element (yellow), and the 2 downstream activating elements (grey and pink) in the putative major late promoter region of porcine adenovirus serotype 5 (species *Porcine mastadenovirus C*) strains HNF-70, GES7 and Z11. The virus serotype (isolate)/GenBank accession number are shown for PAdV-5 reference strain HNF-70, whilst the virus name/host/country/year have been mentioned for strains GES7 and Z11. A '*' denotes an identical nucleotide (nt) residue. The nt positions correspond to those of the complete genome sequence of strain HNF-70.

```
                          5055                                                        5114
PAdV-5_(HNF-70)/AF289262     CGTCATCGTCTTCCACATCTAGAAATGTGATTGGTTTATATTTGTATGTCACGTGATCAG
GES7/Pig/DOM/2020            CGTCATCGTCTTCCACATCTAGAAATGTGATTGGTTTATATTTGTATGTCACGTGATCAG
Z11/Pig/DOM/2021             CGTCATCGTCTTCCACATCTAGAAATGTGATTGGTTTATATTTGTATGTCACGTGATCAG
                             ************************************************************

                          5115                                                        5174
PAdV-5_(HNF-70)/AF289262     ATTTGCCTATAAAAGGCGTGGCTTCTTCGGCGGCCTCATTGTCCTCGTTGGAGACGTCGG
GES7/Pig/DOM/2020            ATTTGCCTATAAAAGGCGTGGCTTCTTCGGCGGCCTCATTGTCCTCGTTGGAGACGTCGG
Z11/Pig/DOM/2021             ATTTGCCTATAAAAGGCGTGGCTTCTTCGGCGGCCTCATTGTCCTCGTTGGAGACGTCGG
                             ************************************************************

                          5175                                                        5234
PAdV-5_(HNF-70)/AF289262     CGACAGGTGGGTAGGCTACGACAAATTCTGGCATGATGTCCGCGCTGAAATTGTCTGTCT
GES7/Pig/DOM/2020            CGACAGGTGGGTAGGCTACGACAAATTCTGGCATGATGTCCGCGCTGAAATTGTCTGTCT
Z11/Pig/DOM/2021             CGACAGGTGGGTAGGCTACGACAAATTCTGGCATGATGTCCGCGCTGAAATTGTCTGTCT
                             ************************************************************

                          5235                                                        5294
PAdV-5_(HNF-70)/AF289262     CTATGAAGGATGAAGATTTGACAGAGTACTTGCCCCTGGAGATGTTCTTTGAGCTCTCTT
GES7/Pig/DOM/2020            CTATGAAGGATGAAGATTTGACAGAGTACTTGCCCCTGGAGATGTTCTTTGAGCTCTCTT
Z11/Pig/DOM/2021             CTATGAAGGATGAAGATTTGACAGAGTACTTGCCCCTGGAGATGTTCTTTGAGCTCTCTT
                             ************************************************************
```
