# Supplementary material for: High Rates of Detection and Molecular Characterization of Porcine Adenovirus Serotype 5 (Porcine mastadenovirus C) from Diarrheic Pigs
Source: Pathogens. 2022 Oct 20;11(10):1210. doi: 10.3390/pathogens11101210 (PMC9610507; doi:10.3390/pathogens11101210)
Supplement: Supplementary file 1 [file pathogens-11-01210-s001.zip › Supplementary table S2.pdf]

Supplementary table S2. Primers used in PCR assays to obtain the full-length putative DNA-dependent DNA polymerase (pol) and hexon coding sequences of porcine adenovirus serotype 5 (PAdV-5, species *Porcine mastadenovirus C*) strains from the Dominican Republic. Additional primers (designed from the obtained nucleotide (nt) sequences) used in the sequencing reactions are not shown. Primers employed in the PAdV-5-specific semi-nested PCR screening assay are highlighted with green.

**Primer name**	**Primer sequence [1] (5′-3′)**	**Primer position [2]**
Pol-PCR-1-F	GTGGTCAAAAGAGGTAAACATTTGG	nt 4205-nt 4229
Pol-PCR-1-R	CCTTCATAGAGACAGACAATTTCAGC	nt 5243-nt 5218
Pol-Semi-Nested-R	CTTAGGGCCAAGGGACACGC	nt 4617-nt 4598
Pol-PCR-2-F	CAGACACACGTCATCGTCTTCC	nt 5047-nt 5068
Pol-PCR-2-R	AGGAATCATACCAGTCGTTCATCG	nt 6098-nt 6075
Pol-PCR-3-F	CAGATGTCATACACGTAGATGGGC	nt 5825-nt 5848
Pol-PCR-3-R	TAGCCCAGGATCTGAACTGGTG	nt 6920-nt 6899
Pol-PCR-4-F	TTGGACTGGTGACAAATCACCTG	nt 6578-nt 6600
Pol-PCR-4-R	CACGCTGAATTATCTCCACCAC	nt 8040-nt 8019
Pol-Nested-PCR-4-F	CGGCCAGAACAATCTCATCAAAG	nt 6603-nt 6625
Pol-Nested-PCR-4-R	GAGATATGTCATGCTTAACCTGG	nt 7992-nt 7970
Pol-PCR-5-F	AGCCGCTGTTTACAACACCC	nt 11730-nt 11749
Pol-PCR-5-R	ACTCTCGTAAGAGGGAGGAGG	nt 12258-nt 12238
Hexon-PCR-1-F	GGATGGCAGGGAACCCTGAATAG	nt 16324-nt 16346
Hexon-PCR-1-R	TTCTGTCCTGCAAGTCAACAACG	nt 17423-nt 17401
Hexon-PCR-2-F	GCTCCTGACACACATCTTGTGTAC	nt 17213-nt 17236
Hexon-PCR-2-R	TCAACATGGCCTCCAGAGTAGAAG	nt 18221-nt 18198
Hexon-PCR-3-F	GGCAGATACTGCCAGTTTCACATC	nt 17975-nt 17998
Hexon-PCR-3-R	TAGGAAACCTGGAAACCGGTTGTC	nt 19278-nt 19255

[1] The primers were designed from the complete genome sequence of PAdV-5 strain HNF-70 (GenBank accession number AF289262).

[2] Nucleotide positions are those of PAdV-5 reference strain HNF-70 (GenBank accession number AF289262).
